# Hawaiian geothermal fumaroles contain diverse and novel viruses

**DOI:** 10.1128/spectrum.01567-26

**Published:** 2026-07-15

**Authors:** Pia Sen, Lausanne Lee Oliver, Kira S. Makarova, Yuri I. Wolf, Christina Pavloudi, Max Shlafstein, Jimmy H. Saw

**Affiliations:** 1 Department of Biological Sciences, George Washington University 166723, Washington, DC, USA; 2 Division of Intramural Research, National Library of Medicine, National Institutes of Health 2511, Bethesda, Maryland, USA; 3 European Marine Biological Resource Centre (EMBRC-ERIC) 577318, Paris, France; Universidad Tecnica de Ambato, Ambato, Ecuador

**Keywords:** geothermal, volcanic, fumarole, bacteriophages, biofilms, biogeography

## Abstract

Viral community structure is known to influence the evolution of microbes in diverse and complex environments. While the diversity of microbes and their viruses have been metagenomically explored in terrestrial hot springs and hydrothermal vent systems, other volcanic features remain remarkably understudied. Fumaroles (steam vents) are geothermal features that heat groundwater with magma, releasing heated water vapor and volcanic gases, such as CO_2_ and H_2_S. Fumaroles are physicochemically dynamic compared to terrestrial hot springs—temperatures and gas emissions fluctuate rapidly with volcanic activity. The viral community structures and diversity have never been systematically characterized or explored. We hypothesize that viruses facilitate microbial community adaptation to the harsh and dynamic fumarole environment. Using a sensitive profile-based approach for identification, we identify 383 viral operational taxonomic units (vOTUs) from 46 metagenomes of biofilms hosted near basaltic fumaroles. We estimate two previously undescribed order-level clades of Caudoviricetes (tailed phages), and find evidence of phylogenetic diversification within the fumarole systems. Read-mapping analysis of three sampled geothermal regions shows unexpected diversity and community structure within the geologic system: 99.7% of fumarole vOTUs are shared between distant fumaroles, and 40°C–60°C biofilms have high viral richness and evenness that do not correspond to biofilm microbial composition or diversity. Lastly, we provide the first description of a terrestrial environment dominated by Microviridae, which has only been described in viral communities of deep-ocean hydrothermal vents. Our study offers a unique geological system for the exploration of viral ecology in extreme environments.

**IMPORTANCE:**

Geothermal environments serve as natural laboratories for studying adaptations to extreme conditions that challenge the limits of microbial life and offer insight into early life on Earth. Exploring microbial diversity in these systems reveals how ecological factors shape complex communities in extreme environments. Evidence increasingly shows that viruses influence microbial diversity in terrestrial hot springs and oceanic hydrothermal vents, yet the biogeography of viruses across these systems remains largely unexplored. We present the first metagenomic characterization of viral diversity and ecology in Hawaiian terrestrial volcanic fumaroles. Our results indicate extensive viral dispersal, in contrast to the typically more constrained dispersal observed in hot springs and hydrothermal vent systems. Furthermore, we observe a dominance of ssDNA viruses in fumarole viral communities, a pattern not previously reported in terrestrial systems. Our comprehensive analyses indicate that Hawaiian fumaroles are a valuable system for studying community patterns and the ecological determinants of viral biogeography.

## INTRODUCTION

Fumaroles (terrestrial steam vents) are the most common, yet least explored geothermal feature on Earth. Fumaroles are formed when steam and volcanic gases exit cracks in basalt deposits (1). Temperature and volcanic gas emissions fluctuate with volcanic activity and geological context (2). Located on the volcanically active Big Island of Hawaii, the East Rift Zone (ERZ) is a geologically active region in the vicinity of Hawaii Volcanoes National Park. Along Hawaii Route 130 and between the towns of Pahoa and Kalapana, there are many physicochemically diverse steam vents, which produce steam that is mainly composed of water. These fumaroles occur on two lava flows with a wide age range (64–400 years old) and host phototrophic biofilms dominated by *Cyanobacteria*, *Chloroflexota*, *Deinococcota*, and *Alphaproteobacteria* (2). Previous investigation of these fumaroles reveals microbial succession patterns that are dependent on early colonizing phototrophs, with Actinobacteria and Proteobacteria becoming more abundant in the biofilms as geothermal activity decreases in the system (2, 3). Functional annotation of microbial taxa from fumaroles suggests that photosynthesis, CO_2_ fixation, and central carbon metabolism are the primary biogeochemical processes in the biofilms. The presence of taxa associated with nitrogen and sulfur cycling indicated these processes may be relevant to biofilm biogeochemical cycling as well. Metals and minerals are made bioavailable in fumaroles by microbial alteration of basaltic substrate, while CO_2_ and H_2_S emissions from the vent stimulate carbon fixation and sulfur oxidation (1).

Microbial viruses modulate functional microbial diversity through one of three main actions: top-down predation of microbial hosts, transduction, or metabolic rewiring during infection, which contributes to metabolic reprogramming of hosts on an ecosystem-level scale (4, 5). The Bank model of viral ecology describes ecosystems where a fraction of viruses are active and abundant, while a myriad of other viruses are inactive and present in low abundances until a shift in environmental conditions (e.g., seasonal or diel intervals) gives rise to new dominant host-virus pairs, as observed in the succession patterns of environmental viral populations (6–8). The Bank model predicts that a viral community high in viral operational taxonomic unit (vOTU) richness will be low in vOTU evenness. The density-dependent “Kill-the-Winner” paradigm describes virulent viruses infecting the most abundant or fastest-growing hosts, while the “Piggyback-the-Winner” paradigm describes temperate viruses increasing in abundance with rates of lysogeny (9). While relative abundance cannot be taken as confirmation of viral activity, it is a metric that provides a good indicator of active viral populations (8, 10).

Biogeography offers a framework for explaining evolutionary patterns by characterizing the taxonomic distribution of microbes across space. While microbial dispersal has previously been considered functionally stochastic, increasing evidence suggests that microbes and viruses exhibit biogeographic patterns (11). These biogeographic patterns are particularly evident in extreme environments, such as geothermal hot springs and hydrothermal vents, where harsh selective pressures limit the presence of higher-order life (12, 13). The exclusion of non-microbial life makes geothermal systems an ideal model for elucidating local adaptations of complex microbial communities, providing insight into how viruses can mediate the genome evolution of microbes in different environmental contexts.

Previous metagenomic studies of geothermal systems, including hydrothermal vent and hot spring fields, indicate that microbial and viral communities are distinct across these environments, and that individual geothermal features (such as vents or pools) are dominated by endemic microbes and viruses, with few shared viruses between features within a geothermal field (11, 13–17). These conclusions are based on viral (and microbial) distance-decay, as well as strong habitat clustering observed in metagenomic studies of viruses across diverse geothermal systems with broad ranges of temperature and physicochemical conditions (14, 15, 18–20). The inconsistent geographic scales upon which these distinct communities are observed suggest that geologic system-dependent dispersal limitation plays a role in determining the community structure of viruses. Microbial mats (biofilms) in geothermal hot springs are particularly low-mobility environments, with highly specific host-virus pairs formed, and low gene flow observed between geographically distant hot spring pools (12, 14, 15).

We explored the structure of viral communities in Hawaiian fumarole biofilms from three geothermal sites within the East Rift Zone: the steam pit (heated by steam rising from the main pit, as well as smaller crevices), the steam wall (epilithic biofilms heated across exposed rock walls), and epilithic biofilms heated from features on the walls and floors of Big Ell cave in the Kilauea Caldera (21). The microbial diversity of steam vent fumaroles is strongly shaped by both interspecific competition and habitat filtering. Similar to hot spring microbial communities, fumarole microbial communities function as ecological islands with limited gene flow between thermal features within the same geologic system (2, 13, 15). However, viral community structure has never been investigated in a fumarolic geologic system.

First, we ask what viral community structures (viral taxa and abundance) are observed across fumaroles in the three sampled geothermal fields. We hypothesize that increasing geographic distance between geothermal features will correspond to fewer shared viruses, with relative abundance decreasing as distance increases. We will observe very few shared vOTUs between the steam pit and steam wall (~100 m distance between fields) and even fewer shared vOTUs between these fumaroles and vent features in Big Ell cave (~35 km from the steam pit and wall). If there are indeed a few shared viruses between fumaroles, we expect that there will be very little evidence of gene flow between viruses and with hosts.

Second, we ask what relationships exist between microbial and viral community structure and diversity. We hypothesize that viruses may facilitate adaptation to the harsh and dynamic fumarolic environment, and that resulting viral community structures will contain a seed bank of highly host-specific viruses characterized by high richness and low evenness, with few highly abundant taxa. We predict that viral and microbial diversity will be directly proportional, such that more diverse microbial communities support a larger viral seed bank. Furthermore, we predict that there will be auxiliary metabolic genes (AMGs) that indicate viral-mediated adaptation of microbes to fumaroles.

We seek to characterize fumarole virus communities by metagenomically identifying viruses using a custom approach that is sensitive and profile-based, and assess viral novelty through network-based analysis and phylogenetic estimation. To test our hypotheses of viral community structure, we utilize read mapping of vOTUs and relative abundance fluctuations across samples. We characterize viral-mediated microbial adaptation through genome annotation of viral metabolic genes and identify evidence of inter-viral competition via superinfection exclusion and viral-encoded defense-related genes (22). We predict the presence of tightly coupled host-virus pairs with multiple unique CRISPR spacer matches within a metagenome-assembled genome (MAG) to a single vOTU, and search for corresponding fluctuations in abundances of viral and microbial populations across samples (23). Our study provides insight into the diversification of early life and patterns of viral and microbial succession in a novel geologic system (24, 25).

## MATERIALS AND METHODS

### Sample collection and sequencing

Detailed methods on sample collection and sequencing have been described in two previous studies (2, 21). Briefly, a total of 46 biofilm and soil samples were collected in an expedition in 2019 from steam vent features found within Kilauea Caldera inside Hawaii Volcanoes National Park and within the East Rift Zone along Hawaii Route 130. DNA from samples was extracted using the ZymoBIOMICs mini prep kit, quantified with Qubit HS dsDNA assay, then sent for sequencing at two sequencing centers: ASGPB at the University of Hawaii at Manoa for 16S amplicon sequencing and CQLS at Oregon State University for whole-genome shotgun (WGS) sequencing using Illumina MiSeq and HiSeq, respectively. Some of the amplicon data have been analyzed in a previous study that excluded the cave soil samples from Big Ell (2). BBTools script bbduk.sh (26) was used for quality control prior to assembly. Adapters were trimmed from the right ends of the reads, with reads shorter than 50 bp discarded (--minlen=50). Adapters were trimmed based on paired overlap (tbo=t) with low-quality bases trimmed on both ends of reads (--qtrim=rl) with low-quality bases discarded (--trimq=20).

### Identifying viral sequences

The ViralAssembly module of metaviralSPAdes v3.15.5 (27) was used for viral assembly and mismatch correction (k-mer lengths of 21, 33, 55, and 77). Assembled contigs were input into ViralVerify v1.1.0 (27) (Naive Bayesian classifier on hmmsearch gene predictions), geNomad v1.11.2 (28) (hybrid approach of alignment-free models and gene models with neural networks for classification), and VirSorter2 v2.2.4 (29) (multi-classifier with HMM search for viral hallmark genes) for initial identification. These tools employ a diversity of methods for viral identification (27–30). Viruses were then identified by identifying both a head and packaging gene in each candidate viral contig. Default parameters were used for ViralVerify with the “-hmm” flag. geNomad was run with the “--end-to-end” flag. As per community standards for VirSorter2, the default score of 0.5 (low-cutoff) was used for maximum sensitivity and filtering. CheckV v1.0.3 (31) was then used on extracted contigs (end_to_end option) to remove false positives and trim host regions of proviruses prior to running VirSorter2 again on trimmed sequences (4, 29). Lastly, for the VirSorter2 pipeline, a secondary screening approach was employed on domain architecture DRAM-v annotations searched for capsid-associated proteins (Phage-coat PFAM clans CL0373 or CL0371). PFAM clans account for evolutionary relationships that cannot be encompassed by a single HMM profile of DRAM-v (32, 33).

Open reading frames (ORFs) were called in metagenomic (“-meta”) mode with Prodigal v2.6.3 (34). Gene calls were annotated with HHblits (35) (uniprot_sprot_vir70 database) with flag “-n 3” iterations for remote homology prediction. Annotations were filtered for E value (<1E-1) and probability (>50%). The first 10 hits for each coding DNA sequence (CDS) were screened for head and packaging-associated genes using an automated Python script, which iterated over annotations by contig. A list of 33 gene ontology (GO) terms identified as head or packaging genes was curated from the QuickGO display (36). UniProtKB annotations that correspond to GO terms for virion assembly and maturation were extracted and curated to exclude baseplate and tail gene products (37). The resulting list of 332 UniProtKB codes was used for the Python search script, which flagged contigs containing both head and packaging-associated gene products, which were then manually verified. All manual checks for gene functional prediction were performed with a combination of BLAST (38), UniProt BLAST, and HHPred (Swissprot DB) (39).

### Viral clustering

Viral sequences were also grouped into roughly genus-level viral clusters (VCs) using vConTACT3 v.3.0 with database (v220) domain restricted to “prokaryotes” (30). Graph output (.cyjs) was imported into Cytoscape (v3.10.2), and the NetworkAnalyzer plugin was used to evaluate the quality of clusters (40). “Clustering coefficient” and “topological coefficient” metrics were filtered to be equal to or greater than 0.85 (4). Subclustering (virus cluster of three or more nodes without links to the rest of the network) is observed for 20 VCs. Packaging and head-related genes of viruses that were unable to be taxonomically assigned into a realm by vConTACT3 v.3.0 were manually verified using BLAST, UniProt BLAST, and remote homology prediction tool HHPred. Genes for major capsid protein and TerL subunit were annotated (HHblits) and clustered (BLAST 2.14.1+) to assess cluster success with viral sequences of variable length (variation >1 kb).

### Generating vOTUs

Viruses were clustered into species-rank vOTUs reflecting viral populations using Minimum Information about an Uncultivated Virus Genome (MIUViG) standards with recommended thresholds (95% ANI and 85% AF) for clustering parameters (41). Contigs were first compared with nucleotide BLAST (v2.11.0+) and pairwise comparisons were generated by blasting all sequences together (“-outfmt ‘6 std qlen slen’ -max_target_seqs 10000”). Finally, clustering of viral sequences was carried out using scripts anicalc.py and aniclust.py (“--min_ani 95 --min_tcov 85 --min_qcov 0”), which are distributed as part of the CheckV package (31).

### Calculating vOTU diversity and abundance

Individual viral contigs were mapped using Bowtie2 (42) with the bowtie2-build function to create .sam files. The .sam files were converted to .bam files, then sorted, indexed, and counted (samtools idxstats) with SAMtools (43). Relative abundance was calculated with reads per kilobase per million mapped reads (RPKM) for each viral contig and then summed for all contigs for each vOTU (44). vOTU richness and evenness were calculated with the vegan package v2.7-2 (45) in R. Richness was assessed for each sample by finding the Chao1 index using the sample vOTU relative abundance table, which was calculated with the specnumber() function. Shannon diversity was calculated for each sample with the diversity() function from the sample relative vOTU abundance table. Pielou’s evenness was calculated by dividing the Shannon index by the ln() of Chao1. The relationship between the temperature of samples and vOTU diversity indexes was assessed for significance with a non-parametric least squares regression, and then assessed for overfitting with a Bayesian information criterion test with sample correction (BICc) due to sample size (*n* = 20). Linear, quadratic, and cubic models were attempted, and the lowest BIC value indicated the best-fit model. Normal distribution and constant variance of residuals were supported with density and QQ-plots for both relationships (Fig. S7). Normality for models of temperature and related diversity indexes was confirmed with the Shapiro-Wilk test.

### Phylogenetic classification of viruses

Phylogenetic estimation was guided by vConTACT3 v.3.0 (30, 46). Phylogenies were estimated using maximum likelihood independently for classes Caudoviricetes, Malgrandaviricetes, Laserviricetes, and Tectiliviricetes. Caudoviricetes references were chosen by selecting first neighbors (“first neighbors of select nodes” Cytoscape v3.10.2) of vConTACT3 v.3.0 assigned fumarole Caudoviricetes (47). For Malgrandaviricetes, Laserviricetes, and Tectiliviricetes phylogenies, all known references were selected from ICTV MSL40.v1, and ORFs were called with Prodigal v2.6.3 (34). All references were annotated as described above with HHblits to identify marker genes. The marker genes were TerL, major capsid, and packaging ATPase marker genes for the Caudoviricetes, Malgrandaviricetes, Laserviricetes, and Tectiliviricetes trees, respectively, with 1,000 iterations of tree topology, branch lengths, and substitution model parameters (48,49). Trimming of the multiple sequence alignment was performed with trimAl v1.5.rev0 with a gap threshold of 30% (50). IQTree v2.4.0 was used for phylogenetic reconstruction, with the evolutionary model Q.pfam+F+I+G4 selected as the best model for *Caudoviricetes* (51). The model Q.pfam+G4 was used for Malgrandaviricetes and *Laserviricetes* trees, and the Q.pfam+I+G4 model was used for *Tectiliviricetes*. One thousand bootstrap estimates were used for branch support estimation. All phylogenies were midpoint rooted and visualized with iTOL v7 (52)

### Host matching with CRISPR spacers

Putative host prediction was performed with CRISPR spacer matching. CRISPR-Cas systems were identified from the binned nucleic acid sequences with <5% contamination using the standalone tool of CRISPRCasFinder as a Singularity image (53). High-confidence arrays were identified by using “-levelMin 3” parameter. Arrays adjacent to Cas systems within 10,000 base pairs (bp) were identified with the “-ccc” parameter. Spacers were extracted from high-confidence arrays using minCED and clustered with a cutoff of 100% identity using cd-hit-est (directionality assessed with parameter “-r 1”) to dereplicate spacer sequences (54, 55). Targets with host matches were sorted into three categories of mobile elements during the viral identification workflow: plasmid, viral, or unknown mobile genetic element (MGEs). MGEs that did not have chromosomal, plasmid, or viral indicators (structural genes) were characterized as other MGEs. Chromosomal fragments were confidently excluded by scanning matches and eliminating targets with either entire arrays or multiple shared spacers and neighboring sequences, which were marked as “suspicious” matches (Table S7). Viruses identified as viral and plasmid hits or uncertain viral and plasmid sequences (ViralVerify, VirSorter2, and geNomad) were aligned with BLAST (2.14.1+). Matches were filtered for ratio *nident* (identical bp matches) to *qlen* (spacer length) >0.89 to be considered operationally valid.

Matches that could be plasmid-encoded arrays or chromosomal fragments were eliminated as follows (56): CRISPRDetect was used to identify arrays in chromosomal arrays and target elements, which were then aligned with BLAST (2.14.1+). Identical matches between chromosomal and plasmid arrays were discarded immediately. If there were multiple shared spacers and neighboring sequences (spacers and repeat elements), the hit was designated as suspicious (Table S9). Target sequences were searched for ORFs using Prodigal (v2.6.3), and .faa (amino acid sequence) and .sco (ORF coordinates) output files were used in conjunction with BLAST (2.14.1+) match coordinates to identify which ORF was targeted by spacers. Targeted ORFs (CDS or non-coding ORFs) were annotated using BLAST, UniProtBLAST, and HHPred.

### Annotation of viral metabolic and accessory genes

Three categories of genes were systematically recovered from vOTUs: AMGs that may have a role in host metabolism during the infection cycle, viral resistance genes, and viral defense system-related genes. Viral resistance and defense genes are non-essential accessory genes (morons) that provide an adaptive advantage to either the phage or host (57). Annotation for functional gene product prediction was performed with DRAM-v v0.1.2 (58) on all viral sequences. Final vOTUs were processed with VirSorter2 v1.0.1 using the -prep-for-dramv flag, then annotated with DRAM-v to identify viral metabolic genes. Gene curation was performed by filtering and manually scanning both the DRAM-v AMG summary (275 gene predictions) and total annotation output (20,666 genes) for AMGs and accessory genes. Gene product annotations were discarded for genes that did not have an auxiliary score <4 or had DRAM-v auxiliary flags of (V) viral structural component, (A) attachment, (P) viral-specific MEROPS peptidase, or (F) “near end of contig” (4, 59). To eliminate the candidate genes within host-like gene operons (possible miscalled proviral ends), all candidate genes were scanned for flanking viral genes using the DRAM-v flag (V) for structural virion components, annotations such as portal, tail, baseplate, capsid, and structural or replicative genes with VOGDB flags (Xs;Xr). This left 212 putative AMG candidates (Table S8).

To be conservative in AMG prediction, secondary manual curation was performed on putative AMG candidates to eliminate genes that could be involved in viral life cycle processes (59, 60). This led to the removal of putative AMGs involved in nucleotide or amino acid metabolism (DRAM-v and KEGG-MAPPER pathways and modules). Putative AMGs were also discarded if their annotation was overly general (genes annotated as glycosyltransferases, peptidases, glycosylhydrolases, adenylyltransferases, methyltransferases, and ribosomal proteins). Attempts were made to obtain a more specific gene prediction with BLASTp before discarding (59, 61). To predict accessory genes, a secondary manual curation was performed by searching non-metabolic annotations that did not have the (M) auxiliary flag in the DRAM-v master annotation spreadsheet output. Participation in life cycle pathways was assessed using methods described above, and resulting accessory gene candidates were manually inspected.

Prophage annotation and boundaries were performed by aligning viral contigs to MAGs with BLAST+ alignment (2.14.1+). Prophage boundaries were identified in viral contigs PHASTEST and manually checked for flanking viral genes on viral contigs using annotation methods described in the workflow for viral identification (62). ORFs were called with Prodigal (v2.6.3) and annotated with Pharokka (v1.7.5), identifying phage orthologous groups (PHROGs) with local alignment (63). Gene diagrams (genomic context determined with MMSeq2 and pyHMMer) were plotted using LoVis4u (64, 65). Proteomic similarity matrix for *Microviridae* viruses was built with MMSeq2 alignments through LoVis4u.

## RESULTS

### Sampling and retrieval of viruses from metagenome

Biofilm samples from ERZ fumaroles are described in previous studies (2, 21). Briefly, biofilms that were sampled were several centimeters thick and grew on the surface of volcanic rock, directly where steam was being released. These biofilms do not exhibit clear segmentation of layers as observed in some microbial mats and display a variety of phenotypes and colors (21). Most Gloeobacter-dominated biofilms are purple with patches of dark green, while other biofilms are green, yellow, brown, and gray. While some biofilms have a visible extracellular polysaccharide matrix layer and appear wet, other biofilms are dry or otherwise textured in appearance. While the steam pit and steam wall are separated by ~100 m, the Big Ell cave in the Kilauea Caldera is ~35 km from the steam vents along Hawaii Route 130 (21).

WGS raw sequences from related biofilm features were co-assembled for related features (20 metagenomes from the 46 collected samples) with the ViralAssembly module of metaviralSPAdes, producing 400.15 Mbp (12,253 contigs) of assembled sequence data from the metaviralSPAdes pipeline (21, 27). A total of 112.97 Mbp of the assembled sequence data were generated from Big Ell cave in the Kilauea Caldera (Hawaii Volcanoes National Park) with 17.87 Mbp (452 contigs) of the sequence data from geothermal biofilm features within the cave, and 95.11 Mbp (3,803 contigs) of sequence data from adjacent non-geothermal volcanic soil samples. A total of 243.97 Mbp (6,596 contigs) of sequence data were assembled from exposed steam wall biofilms, and 43.21 Mbp (1,402 contigs) of sequence data were assembled from the steam vents along Hawaii Route 130. From these assembled contigs, the ViralVerify module of metaviralSPAdes identified 754 contigs (6.33 Mbp) as either confidently (*n* = 76) or uncertainly (*n* = 678) viral (Table S1). These contigs were then used in downstream analysis. VirSorter2 (29) identified 119.46 Mbp (*n* = 16,002) of candidate viral contigs. From there, the VirSorter2 contigs (annotated with DRAM-V) were filtered for PFAM clan identifiers for the capsid genes (Phage-Coat: CL0373 and Inovirus-Coat: CL0371), producing 14.29 Mbp of candidate viruses (802 contigs), which were used for downstream analysis.

The accuracy of VirSorter2 filtering by PFAM clan was assessed by screening the remaining 15,200 contigs identified as candidate viruses by VirSorter2 for the terminase large subunit gene (TerL) or another packaging gene (Table S2). Three hundred twenty contigs (~2% of filtered VirSorter2 output) contained TerL and were manually assessed for viral identity, with upstream and downstream regions searched for other viral components. Of these contigs, 116 (0.76% of filtered contigs) were determined to be viral (containing both head and packaging genes). With geNomad (28), we identified 3.41 Mbp (*n* = 107) viral candidates (Table S3). When final viral designations were made, and vOTUs were generated, 24 vOTUs were identified by all of the tools used: VirSorter2, ViralVerify, and geNomad (Fig. S1). Fifteen vOTUs were identified by both VirSorter2 and ViralVerify, and a single vOTU was identified by both ViralVerify and geNomad. The majority of vOTUs (*n* = 313) were identified from VirSorter2. Six vOTUs were only identified by ViralVerify, and 5 vOTUs were identified only by geNomad.

### Remote homology-based identification and network analysis reveal diversifying vOTUs

**Fig 1:**
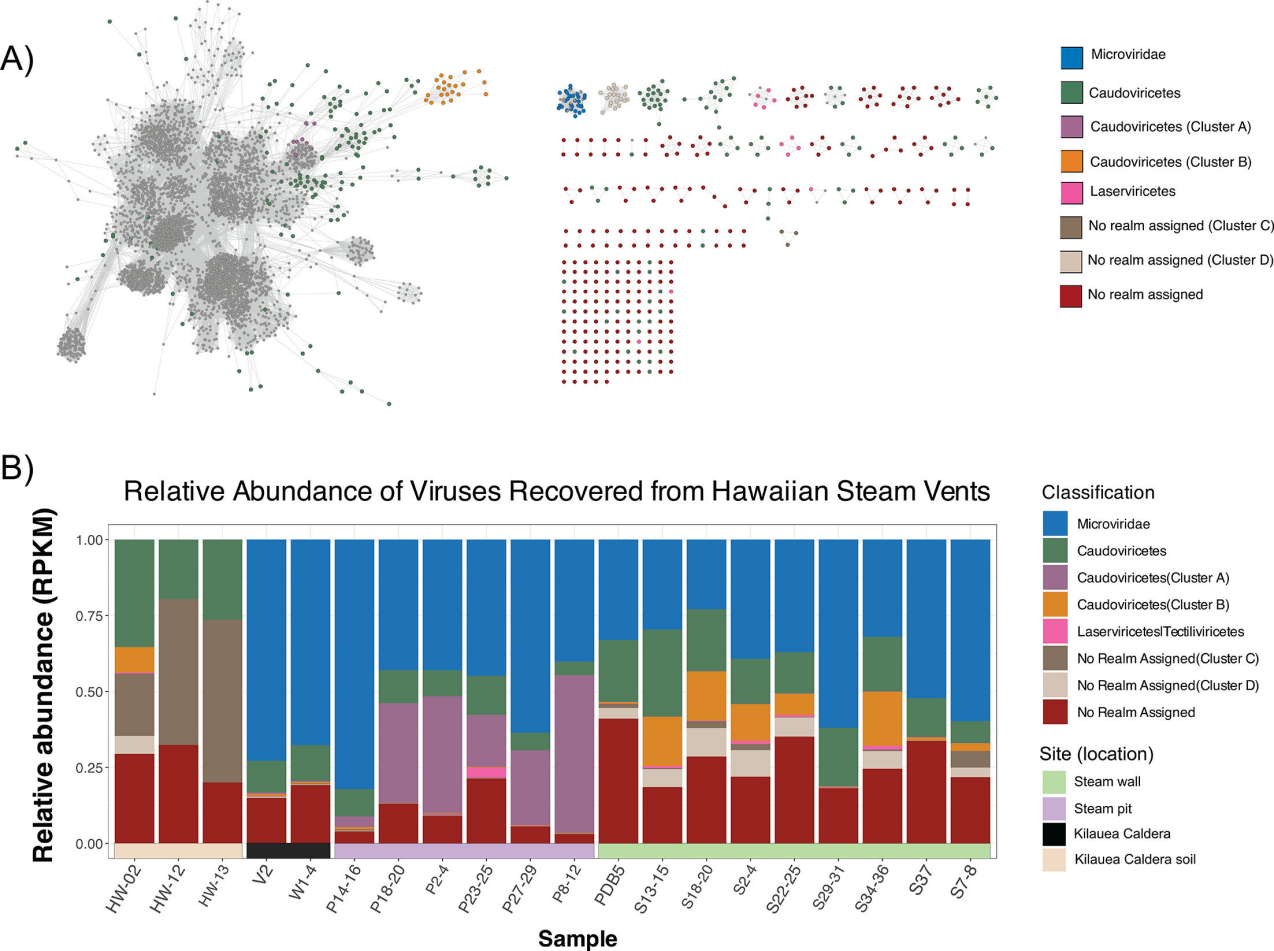
(**A**) Gene-sharing network of confidently identified fumarole viral contigs visualized in Cytoscape. Nodes represent full viral sequences. Gray nodes are RefSeq viruses from the vConTACT3 v220 database, while colored nodes are fumarole viruses, grouped by vConTACT3 taxonomic predictions. Categories indicate the lowest confident taxonomic level assigned to each cluster; highly abundant clusters are colored independently of taxonomy. Edges represent the strength of gene-sharing between viruses, determined with protein clusters. All shown clusters have high clustering coefficients (>0.85), as calculated using the Cytoscape NetworkAnalyzer plugin. (**B**) Relative abundance of identified viruses in all 20 samples, colored by cluster classification. Relative abundance (RPKM) was calculated for each individual viral contig and grouped by the taxonomic designation of clusters for visualization. Clusters found in notable abundance were colored and denoted independently, regardless of predicted taxonomy. Relative abundance of vOTUs used for statistical analysis is available as Table S6.

A total of 569 viral contiguous sequences (contigs) were identified from 20 fumarole sample metagenomes (Table S4). From these contigs, we performed clustering by community standards (41) and sorted viral sequences into 383 species-rank level vOTUs (viral populations). Fifty-one percent of identified fumarole vOTUs are identified as Caudoviricetes (195 vOTUs), indicating that microbial viruses make up a significant portion of the metagenomically retrieved dsDNA viruses in fumarole biofilms. Forty-six percent of vOTUs (180 vOTUs) cannot be placed into realms by vConTACT3 v.3.0 despite verification of structural viral genes on each contig. These unclassified vOTUs may have ecological significance, making up at least 10% of viral relative abundance in the majority (17 out of 20) of samples (Fig. 1).

Of the 180 vOTUs that could not be placed into a realm with network analysis, 100 of the “no realm” vOTUs were missed by geNomad and filtered out in the VirSorter2 pipeline (4) during the CheckV step (31), suggesting stringent pipelines may miss some of the ecologically significant and highly divergent viruses (8, 28). The vOTUs were placed into genus-rank VCs established by vConTACT3 v.3.0 and were visualized in Cytoscape (v3.10.2) with and without all viruses from NCBI RefSeq (66). Clusters were filtered for quality by topological and clustering coefficients (>0.85). This network yielded 230 distinct topological groups, including subclusters, nested subclusters, and singletons within the network (Fig. 1). Of these topological formations, 20 VCs form distinct subclusters (clusters of viral genomes with three or more genomes without links to the rest of the network). Multiple distinct viral populations (vOTUs) within a single VC provide evidence of genomic variation and diversification within the genus (30). High “closeness centrality” values support the relationships observed within network clusters and prevent incomplete viral genomes from inflating network representation of genomic distance between vOTUs (31, 40). Resulting network clusters made up entirely of distinct fumarole vOTUs indicate that related viral populations share many genes with each other while still providing evidence of diversification between related vOTUs.

While 61% of Caudoviricetes clusters contain 1 vOTU, 6 clusters have between 8 and 24 different vOTUs in a single cluster (Fig. 1). One highly abundant cluster, Caudoviricetes (cluster B), makes up to 20% of viral relative abundance in three different biofilm samples from the steam wall. This cluster contains 20 unique vOTUs. The high abundance of related vOTUs is evidence of potential speciation occurring in abundant viral populations (8). There is also diversity observed within VCs of viruses that cannot be placed into a realm confidently by vConTACT3, providing tentative evidence of diversification within highly novel viral genera: out of the 21 VCs (three or more viral genomes within a cluster) which could not be realm placed, 11 of the “no realm” filtered VCs contain more than a single vOTU (Table S5). All of these 11 VCs are internally coherent with high connectivity measured by the NetworkAnalyzer plugin (40).

### Phylogenetic estimation of viral novelty

#### Fumaroles contain new order-level Caudoviricetes clades

**Fig 2:**
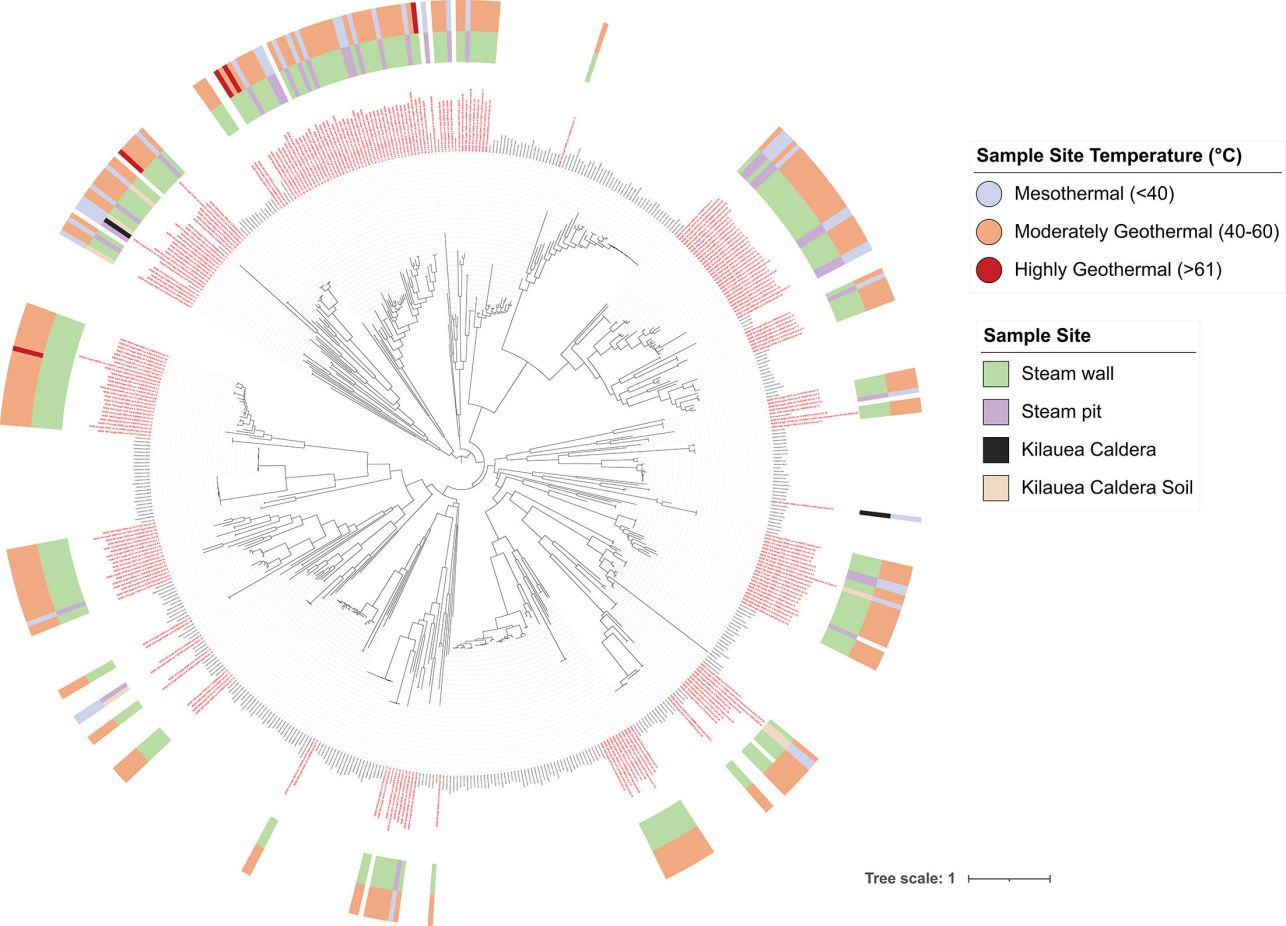
Maximum likelihood phylogenetic tree of Caudoviricetes terminase large subunit (TerL) amino acid gene sequences. Red node labels correspond to TerL sequences annotated in fumarole viral contigs. Black node labels correspond to RefSeq taxon which were determined to be first neighbors of fumarole viruses with the Cytoscape plugin NetworkAnalyzer. Outermost ring refers to the temperature range of the sample from which the virus was metagenomically assembled. The middle ring refers to the sample site from which the corresponding sample was taken. All annotations of TerL in RefSeq and fumarole viruses were made with HHblits using parameters described in Materials and Methods.

Out of the 196 vOTUs identified as Caudoviricetes by VContact3 assignment, 179 Caudoviricetes vOTUs contained a terminase large subunit (TerL) gene, and thus, were included in phylogenetic analysis with maximum likelihood (91% of Caudoviricetes vOTUs). Using 320 first neighbors of fumarole viruses from ICTV MSL40.v1, only 3% of these references could be placed into an existing order. Less than half (40%) of these references could be placed into previously identified Caudoviricetes families. The lack of classification capability for the reference viruses which are most closely related to fumarole vOTUs is evidence of fumarole viral novelty. Many fumarole viruses form distinct clades, with references often paraphyletic or sister taxa to fumarole viruses, suggesting that steam vent viruses expand the diversity of Caudoviricetes. The resulting maximum likelihood tree provides evidence of phylogenetic diversification and potentially speciation within Caudoviricetes clades. There is evidence of recent diversification within at least five clades that are almost entirely made up of fumarole viruses and contain 130 vOTUs (Fig. 2). These species-rank vOTUs meet MIUViG standards necessary for taxonomic proposals (41).

We put forward two Caudoviricetes order-level clades containing 30 and 56 unique fumarole vOTUs, respectively. These clades expand the diversity of dsDNA viruses and enable classification of three previously identified Caudoviricetes families which have not been assigned to orders previously. The first clade, which we call *Kilaueavirales*, contains the *Casjensviridae* and *Herelleviridae* families. The *Casjensviridae* family is made up of flagellotrophic phages which frequently infect Enterobacteriaceae and can be found in high abundances in wastewater (67), while *Herelleviridae* viruses infect the Bacillota phylum of bacteria (68). Clade 1 (*Kilaueavirales*) contains a family-level clade of 30 fumarole virus vOTUs (40 contigs) and 17 previously described RefSeq Caudoviricetes species which had not previously been assigned to viral families. These unassigned reference viruses have hosts such as *Xanthomonas*, *Burkholderia*, and *Enterobacter* (69). This family-level clade of 30 fumarole vOTUs and 20 RefSeq viruses is monotypic with low support (support = 60%) to the *Faecalibacterium* phage, *Eponavirus epona*. This family-level clade and the *Casjensviridae* family together form a well-supported clade (support = 100%) that is a sister group to family *Herelleviridae* (support = 98.9%). Together, this resulting clade is clade 1 (*Kilaueavirales*) (Fig. S2).

Clade 2 (*Pahoavirales*) contains 56 fumarole vOTUs, some of which make up clades that are sister groups to reference viruses (support = 90.5%). One sister group within the *Pahoavirales* clade contains a fumarole virus with a monotypic relationship (support = 89.1%) to RefSeq *Ralstonia*, *Agrobacterium*, and *Myxococcus* viruses. A sister group to this clade contains two sister subclades (support = 95.8%). In one of these sister subclades, there are 24 fumarole virus vOTUs and viruses from the *Mesyanzhinovviridae* family are shallow-branching taxa close to tree tips. The second subclade (which is a sister group to the first subclade containing *Mesyanzhinovviridae*) is mostly made up of fumarole viruses (32 fumarole vOTUs), but also places two reference viruses (*Dinoroseobacter* and *Microbacterium* RefSeq phages) within the subclade (support = 100%). The shallow branching relationships observed in the second sister subclade of *Pahoavirales* suggest recent divergence of closely related viruses without many close relatives (Fig. S2).

In addition to the 86 Caudoviricetes vOTUs which belong to two previously undescribed order-level clades, we observe other clades of Caudoviricetes fumarole viruses with some relation to previously identified viral families (Fig. S3). One fumarole vOTU is estimated to be within the same clade of Caudoviricetes incertae sedis viral family *Hodgkinviridae* (support = 75.1%), which is a family of *Microbacterium* phages (70). We estimate 19 fumarole vOTUs (25 viral contigs) to be a sister group to the *Hodgkinviridae* family (support = 93.7%). Six fumarole vOTUs are a sister group to the *Straboviridae* family (support = 87.8%) (70). Five fumarole Caudoviricetes vOTUs are estimated to form a clade with the incertae sedis *Peduoviridae* family (support = 85.9%). Two fumarole vOTUs are part of a clade with the *Zobellviridae,* which is an incertae sedis Caudoviricetes family (support = 96.5%). One fumarole vOTU forms a clade with the Caudoviricetes incertae sedis family *Saffermanviridae* (support = 95.6%). The relationships of fumarole Caudoviricetes to numerous Caudoviricetes incertae sedis families provide evidence that the inclusion of these viruses in phylogenetic estimation expands the diversity of dsDNA tailed viruses.

#### Estimation of diverse Singelaviria and Varidnaviria

Viruses within the Singelaviria realm (class of Laserviricetes) and the Varidnaviria realm (class of Tectiliviricetes) are assigned to a single class category of “Laserviricetes|Tectiliviricetes” by vConTACT3. Tectiliviricetes phylogeny was estimated with a maximum likelihood tree that was constructed using the packaging ATPase gene for six different fumarole Tectiliviricetes vOTUs (11 viral contigs). Viral species from all five genera within the *Tectiviridae* family were used as references. The resulting phylogeny shows six distinct fumarole Tectiliviricetes clades (Fig. S4). One vOTU is a sister group with weak support (support = 52.5%) to the Alphatectivirus genus, which contains only the two species of PRD1 and PRD4. Alphatectivirus is the only genus within the Tectiliviricetes class that is known to contain plasmid-dependent phages. This vOTU may be a taxon within Alphatectivirus, which is potentially worthy of further investigation when exploring the phylogenetic distribution of related ecological characteristics, such as plasmid dependence (71). Two fumarole Tectiliviricetes vOTUs, which are closely related and have almost identical packaging ATPase sequences to each other on the amino acid level, are a sister group to the clade containing *Deltatectivirus* and *Epsilontectivirus* (support = 93.8%).

An independent phylogenetic estimation with maximum likelihood was performed for Laserviricetes using the packaging ATPase gene. This phylogeny contains a single fumarole vOTU (Fig. S4), which is a sister taxon with strong support to the *Hukuchivirus* genus (support = 100%). The *Hukuchivirus* genus is made up entirely of viruses infecting the thermophilic genus, *Thermus*. This vOTU could potentially be considered a member of a new genus within the family of *Matshushitaviridae*.

#### Microviridae form a poorly resolved clade with phiX174

A class-level maximum likelihood phylogeny of Malgrandaviricetes was built using the amino acid sequences of the major capsid protein to classify the single fumarole *Microviridae* vOTU. The resulting phylogeny produced a poorly resolved clade, resulting in an apparent polytomy (support = 100%) with Enterobacterial coliphage phiX174 (Fig. S5). These fumarole viruses and phiX174 are paraphyletic to the other genera of microviruses in the *Microviridae* family. The high genome conservation of phiX-like viruses (and more broadly, the *Bullavirinae* subfamily) (72) is potentially explanatory of an apparent polytomy with phiX174 with fumarole *Microviridae*, and suggests that phylogenetics is insufficient to capture the diversity of fumarole microviruses.

#### Comparative genomics of abundant Microviridae

A single vOTU of Microviridae was identified, with some genetic variation (subclustering) observed between phylogenetically identical Microviridae genomes of similar length (Fig. 1). The relative abundance of Microviridae ranges from 22% to 82% across samples of varied microbial composition and biofilm phenotype (Fig. 1). In almost half of the biofilm samples, Microviridae makes up over half of the viral community and is most abundant in moderately geothermal biofilm samples, which have overall high viral richness and evenness. The high abundance of Microviridae in moderately geothermal biofilms is surprising because, to our knowledge, there has never been a terrestrial environment reported that displays high abundances of Microviridae. However, abundant Microviridae have been reported from viromic surveys of viruses in hydrothermal vent sediment, as well as benthic sediment (18, 73). Despite the more pronounced prevalence of Microviridae in moderately geothermal biofilms, mesothermal biofilms also display a higher abundance of Microviridae (>28% viral relative abundance) than both other terrestrial environments and mesothermal volcanic soil collected adjacent to geothermal features from the Kilauea caldera, suggesting Microviridae abundance is not simply predictable by measured temperature.

The low abundance of *Enterobacteria* in fumarole biofilms (MAGs) and amplicon sequence variants (ASVs) suggests that these Microviridae have non-Enterobacterial hosts, and that the apparent polytomy of fumarole viruses with phiX174 could be explained by low phylogenetic resolution rather than functional similarity between environmental *Microviridae* and phiX174 (21). Our observation of viruses from the Bullavirinae subfamily in fumaroles is intriguing. While lab models of phiX-like viruses are heavily studied, the subfamily of Bullavirinae is considered extremely rare in nature and occupies a specialist niche as a lytic phage with limited capability for uptake of genomic content (74).

**Fig 3:**
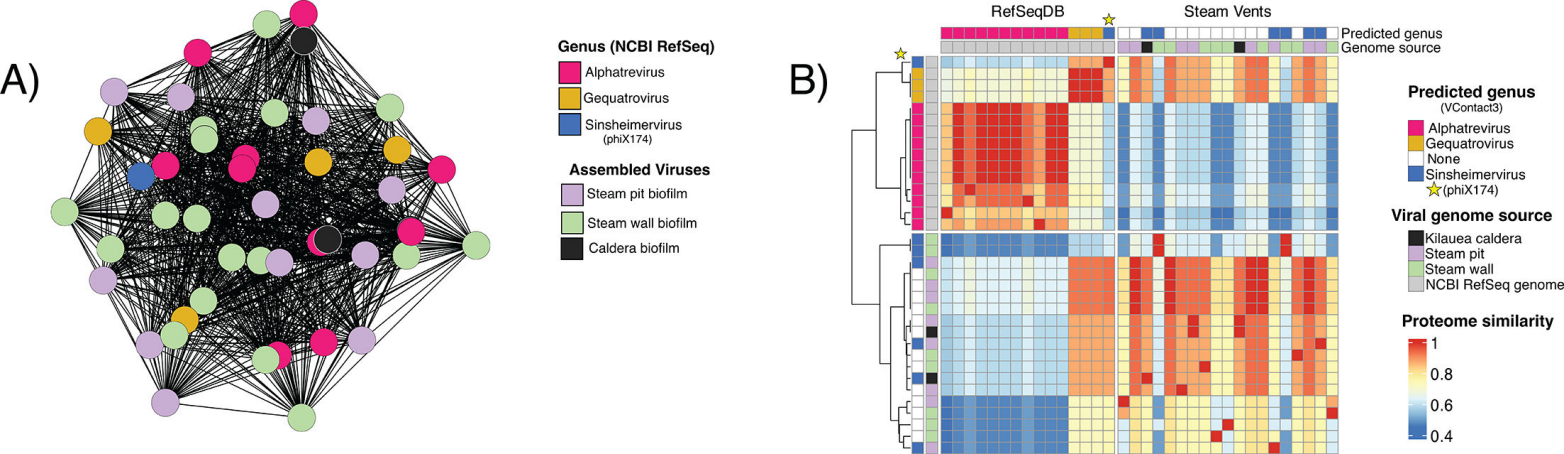
(**A**) Subcluster of viral genomes within the family Microviridae. Network constructed using vConTACT3 and visualized in Cytoscape. Reference nodes are colored by genus (RefSeq and vConTACT3 predictions), while fumarole viruses are colored by sample site. Edges represent gene-sharing strength. The blue node (phiX174) forms a weakly resolved clade with fumarole viruses in the maximum-likelihood phylogeny based on major capsid protein amino acid sequences. (**B**) Pairwise genome comparisons of RefSeq and fumarole Microviridae using local alignment (MMseqs2). The top row and left column indicate predicted genus assignments (vConTACT3) for fumarole and RefSeq viruses. The top-left quadrant shows RefSeq–RefSeq comparisons; the top-right shows fumarole–RefSeq comparisons (RefSeq colored by genus). The second row (genome source) is colored by fumarole sample site. The star denotes comparisons involving the phiX174 RefSeq genome.

Fumarole microviruses were characterized genomically with comparison of whole genomes (amino acid sequences) with RefSeq *Microviridae*. Network analysis with vConTACT3 demonstrates consistency in length of reference and fumarole *Microviridae* (phiX174 genome size is 5.386 Kb, fumarole *Microviridae* are 5.463 Kb), supporting that the potential for genome completion issues does not falsely inflate the degree of genetic difference between fumarole and reference *Microviridae*. Fumarole *Microviridae* are located within the same nested subcluster as viruses from the Bullavirinae subfamily, providing evidence of genetic similarity between fumarole viruses and Bullavirinae viruses beyond simply phylogenetic association. This overall genome conservation may be explained by limited genomic uptake capabilities of Microviridae, which occupies a specialist niche as a lytic phage, constraining genetic rearrangement and mosaicism (72). Despite this, fumarole and RefSeq Microviridae still vary widely, with pairwise similarity as low as 40%–60% between viruses (Fig. 3). In addition to low overall genome similarity, there is evidence of syntenic changes between fumarole Microviridae, with differences from RefSeq Bullavirinae species (Fig. S6).

The low pairwise similarities and syntenic rearrangement we observe between fumarole *Microviridae* are highly unexpected. While innovations do occur in the *Microviridae* genome (gene overprinting, recombination), the overall high syntenic conservation of the *Microviridae* family is thought to reflect strong evolutionary constraints for transcriptional timing of *Microviridae* viruses (72).

### Most vOTUs are shared between biofilms

Hawaiian fumarole microbial communities demonstrate similar selective pressures as hydrothermal vents and hot springs, with endemic and overdispersed microbial compositions suggestive of interspecific competition and habitat filtering (2). These microbial communities have been described as “islands” with distinct community structures across geothermal features. This is similar to microbial community structures hosted by geothermal features across vast physicochemical ranges. From hyperthermophilic hydrothermal vents to moderately geothermal hot springs with significant selective pressure, where both viruses and microbes have been described as endemic communities (12, 13, 15).

In Hawaiian fumaroles, read mapping demonstrates 99.7% of vOTUs identified (*n* = 382) are shared between fumarolic biofilm samples (all but one vOTU). While vOTUs are not often shared between features and fields of hydrothermal vents and geothermal hot springs, it is even more unexpected to observe a high relative abundance of shared vOTUs between geographically distant features. For hot spring and hydrothermal vent systems, viral community dissimilarity increases with geographic distance, and fewer shared vOTUs are observed between distant geothermal fields (regions with localized and relatively high heat flow). We observe 9.5% of fumarolic vOTUs (*n* = 36) are shared between samples with population relative abundance over 1% (Fig. 1). Five percent of identified vOTUs (*n* = 19) are shared between geothermal features and sites in abundances over 2%. The abundance of shared vOTUs between fumaroles is inconsistent with descriptions of distance decay observed in other systems. Though the seed-bank model predicts that most of the shared vOTUs will be found in <2% relative abundance within a given community at any point in time (75), we observe individual fumarole-hosted viral populations that are non-dominant and shared between samples can make up to 10% of a given biofilm’s viral relative abundance, representing a significant variation from the Bank model, even though most vOTUs are not unique to individual fumarole biofilms as predicted by the Bank model (6).

In phototrophic hot spring biofilms with motility limitations, host-virus pairs are tightly coupled and highly abundant (12, 15, 76, 77r). While it is difficult to ascertain microbial and viral dispersal through metagenomics, CRISPR-based analysis provides evidence of host-specific viral coevolution, which is in line with a “Kill-the-Winner” model predicted by the Red Queen hypothesis (76, 78, 79). Ten percent (*n* = 39) of host-matched fumarole vOTUs show evidence of coevolution with hosts (three or more unique spacer matches between a single MAG and vOTU). High numbers of unique spacers between vOTUs and MAGs are observed for an unclassified genus of Rhodomicrobiaceae (*n* = 61), and Chloroflexota families Roseiflexaceae (*n* = 35), and JAJPGT01 (*n* = 34). These spacers are sometimes spread across multiple CRISPR arrays within a single MAG. These arrays are associated with type I and III systems for three Chloroflexota MAGs from novel Chloroflexia and UBA6077 classes (Table S7). This suggests that the multi-spacer matches could be a result of repeated exposure to specific vOTUs or the utilization of multiple CRISPR systems being involved in conferring viral resistance (80). However, these arrays are not necessarily genomically clustered together, with up to ~61 kb observed between these array pairs.

The “Kill-the-Winner” model is usually density dependent and would suggest highly specific pairings between viruses and microbes (9, 15). Despite this, not a single one of the 14 vOTUs which are predicted to be host-linked can be found in relative abundances over 1%. Host phyla and vOTU abundances do not correspond to each other across samples (Table S6) (2, 21). Additionally, these host-specific viruses are not necessarily endemic despite being involved in specialized host-virus pairings (81). While not representative of all microbes in fumarole biofilms, 21% of host-matched MAGs (*n* = 74) appear to evolve closely with viral taxa (21). The inconsistency with both host and viral taxa abundances suggests complex biogeographic factors rather than a simple “Kill-the-Winner” dynamic inform viral and microbial community structure.

While the majority of tightly coupled host-virus pairs are not abundant within the same samples, the exception is for two Caudoviricetes vOTUs (prophages within *Gloeobacter kilaueensis* MAGs). This cyanobacterium (and associated vOTUs) is abundant only in steam pit samples. These Gloeobacter prophage vOTUs range in abundance from 17% to 51% in all but one of the biofilms dominated by *Gloeobacter kilaueensis*. The directly proportional microbial and viral abundances follow the “Piggyback-the-Winner” trend, describing a microbial community in which co-abundant vOTUs favor a switch to lysogeny as a viral life cycle strategy, and accordingly, these abundant viral populations are likely to be prophages (9).

### Viral alpha-diversity

**Fig 4:**
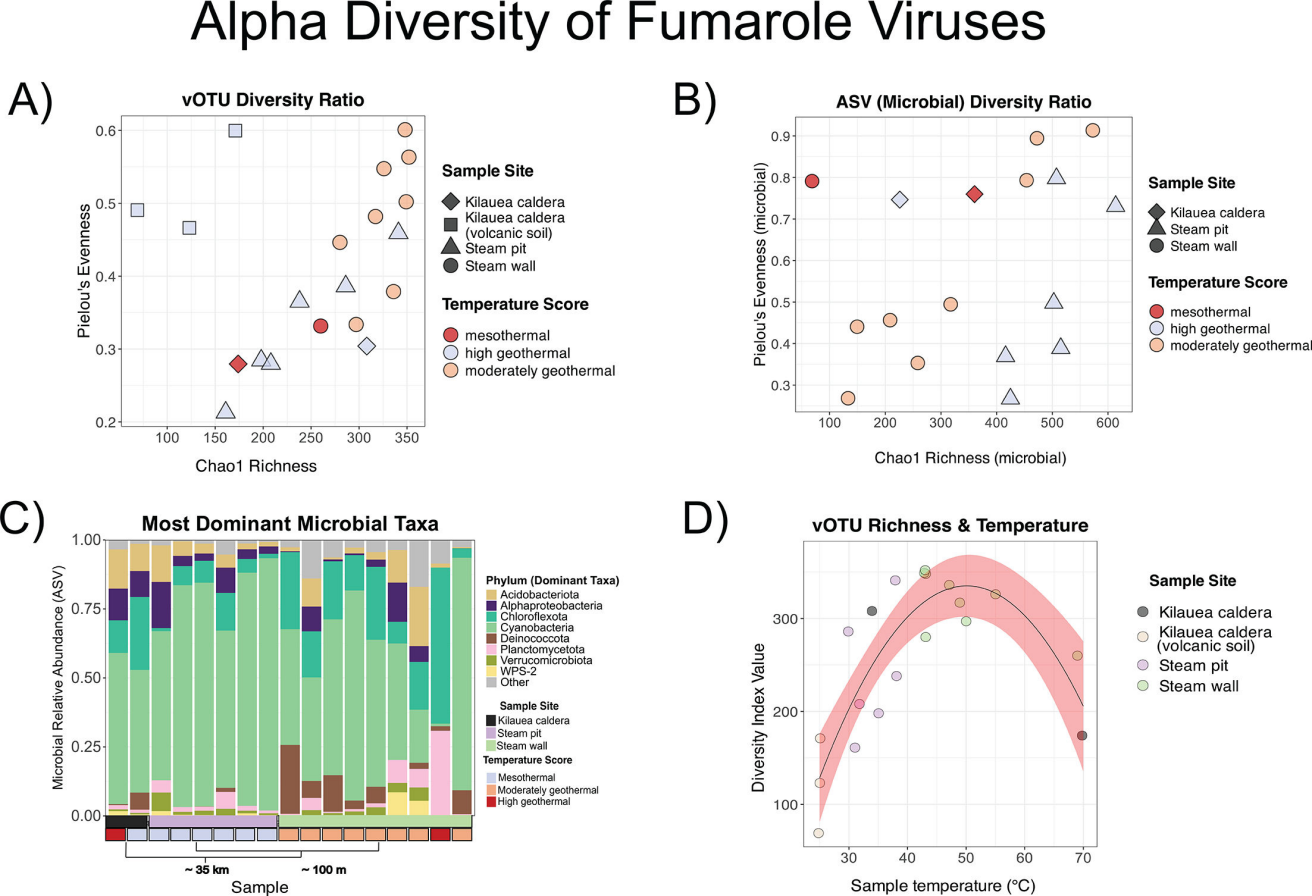
(**A**) Richness (Chao1) and evenness (Pielou) of fumarole vOTUs calculated from RPKM-based relative abundances (Table S6). Each point represents a sample. Shape indicates sample site, and color indicates temperature at the time of collection. (**B**) Richness and evenness of microbial ASV relative abundances. Each point represents a sample; color and shape denote sample characteristics as described in panel **A**. (**C**) Relative abundance of the most abundant bacterial taxa across samples based on ASVs. The top five phyla per sample are shown and used for visualization. The second row indicates sample site, and the bottom row indicates temperature at collection. The phylogram shows spatial relationships among sites: the steam pit and steam wall are ~100 m apart, while the Kīlauea caldera cave is ~35 km from both. (**D**) Sample temperature versus vOTU richness (Chao1). Each point represents a sample, colored by site. vOTU richness follows a temperature-dependent pattern modeled by a polynomial (quadratic) regression. Richness increases with temperature up to ~50°C and declines thereafter.

Chao1 (species richness) and Pielou (species evenness) index values were plotted against each other for each sample (Fig. 4). Moderately geothermal biofilms cluster together, regardless of location or microbial distribution within the sample. Relative to other samples, biofilms that are collected from moderately geothermal fumaroles (40°C–60°C) have high richness and evenness. The 17 thermal biofilm samples show a strong positive correlation between vOTU richness and evenness. On the plot of richness to evenness, non-thermal volcanic soil diversity is clearly partitioned from the fumarolic active biofilms, displaying low vOTU richness and high evenness. The low richness is striking when compared to biofilm diversity because soil has been described as a system that generally has high viral richness and evenness compared to the viral communities of other systems, such as marine and groundwater (59, 82).

Richness and evenness plots exhibit a clear partitioning of diversity (high richness/evenness in moderately geothermal fumaroles) that suggests diverse viral communities may be characteristic of active fumaroles. The biofilms which display this high species richness and evenness are dominated by different microbial taxa, suggesting that microbial taxonomy alone is insufficient to explain viral diversity (Fig. 4). Microbial diversity indexes of biofilms were assessed using ASVs (2, 21). Unlike with viruses, there is no visually evident clustering of temperature-associated diversity (Fig. 4). The decoupling of microbial and viral diversity is observed across samples of varied temperature ranges and microbial compositions. The samples with high viral richness and evenness are composed of diverse phyla (Fig. 4). Interestingly, these dominant phyla all are known to occupy varied niches and display varied metabolic traits, suggesting microbial composition alone is insufficient to be the primary explanatory driver of viral diversity within these biofilms (2).

When the relationship between richness and temperature was assessed, a quadratic model was found to be the best fit to explain this relationship. While the 4-parameter cubic model had the lowest residual sum of squares (RSS) value (RSS = 36,846.4), the quadratic model had the lowest BICc value (BICc = 161.8), indicating that the quadratic model is the best fit for describing the relationship between temperature and viral richness for non-thermal volcanic soil and fumarolic biofilms, ranging in temperature from mesophilic to highly geothermal (>70°C) (2, 21). These tests support the significance of a non-linear (quadratic) relationship between temperature and viral richness in the Hawaiian fumarole system, with the highest vOTU richness observed at ~50°C. The extent to which microbial life adheres to Humboldt’s theory of a temperature-diversity gradient has only been explored in a handful of geothermal environments, with hot springs making up the majority of geothermal systems studied for this (83). In some moderately geothermal circumneutral hot springs, the highest species richness of bacteria has been observed at 50°C, with increasing temperatures associated with decreased species richness (14). While the bacterial diversity of fumarolic biofilms observed does not clearly follow this trend, viral diversity demonstrates a surprisingly consistent temperature-diversity gradient that is independent of microbial diversity (2, 83). This indicates that moderately geothermal features of fumaroles are potential biological hot spots for viruses, with high biodiversity of microbial/viral life that is observed across different geothermal systems at similar temperatures.

The R-squared value (R-squared = 0.7292) indicates temperature explains ~73% of variance in viral richness and is determined to be significant with the *P* value of the F-statistic (*P* = 1.619e-05) (Fig. 4). Normality of residuals was supported by QQ and density plots, and the Shapiro-Wilk test confirms that the quadratic model meets normality assumptions (W = 0.94514, *P* value = 0.2992) (Fig. S7). Though vOTU evenness varies greatly between samples (from Pielou = 0.21–0.6), temperature cannot explain the great variation in viral population evenness (Fig. S7). While there is a significant relationship between vOTU richness and temperature (consistent with the cluster of high viral richness and evenness for moderately geothermal biofilm samples), the evenness of these samples cannot be described as temperature dependent or consistent with any specific microbial phylogenetic distribution, indicating complex biogeographic factors may explain viral diversity of biofilms.

### CRISPR defends fumarole bacteria against a breadth of unknown elements

CRISPR spacer targets (*n* = 302) were identified for arrays from 13 different microbial phyla (Table S7). Matches are found for 20% of all fumarole-assembled MAGs (*n* = 74) (21). The majority of spacer matches (*n* = 120) are for arrays that come from Chloroflexota MAGs (classes: UBA6077, Ktedonobacteria, and Anaerolineae). Despite this, only 70% of the Chloroflexota targets identified are vOTUs (*n* = 9). Additionally, Chloroflexota spacer matches were identified for 1 plasmid (0.65% of all spacer matches) and 33 unknown elements. Beyond the Chloroflexota phylum, spacer targets are not limited to viruses either. Even though the majority of matches identified were to vOTUs targeted by spacers (74.2% of spacer matches), viruses were only 44% of the matched target elements.

These unknown elements represent possible other miscellaneous mobile genetic elements (conjugative/integrative elements, transposons, insertion sequences) (84) or chromosomally integrated elements (85). Even though the majority of spacer targets are non-viral, these unknown element matches make up only 20.86% of total spacer matches. This suggests that the microbial populations of fumaroles utilize CRISPR to defend themselves against a wide breadth of unknown elements, and that CRISPR may play a minimal role in any sustained antagonistic coevolution occurring between non-viral elements and microbes in the fumarole biofilms (86). One exception to this, however, is multiple spacer matches (*n* = 8) between an unknown element and a Chloroflexota MAG (class UBA6077). Seven of these matches to the unknown element are part of a Type IA CRISPR system, while one spacer match is contained within a different array that is part of a Type IE CRISPR system.

### Identification of putative AMGs and accessory viral genes

#### AMG-mediated adaptation to heavy metal toxicity and resource limitation

**Fig 5:**
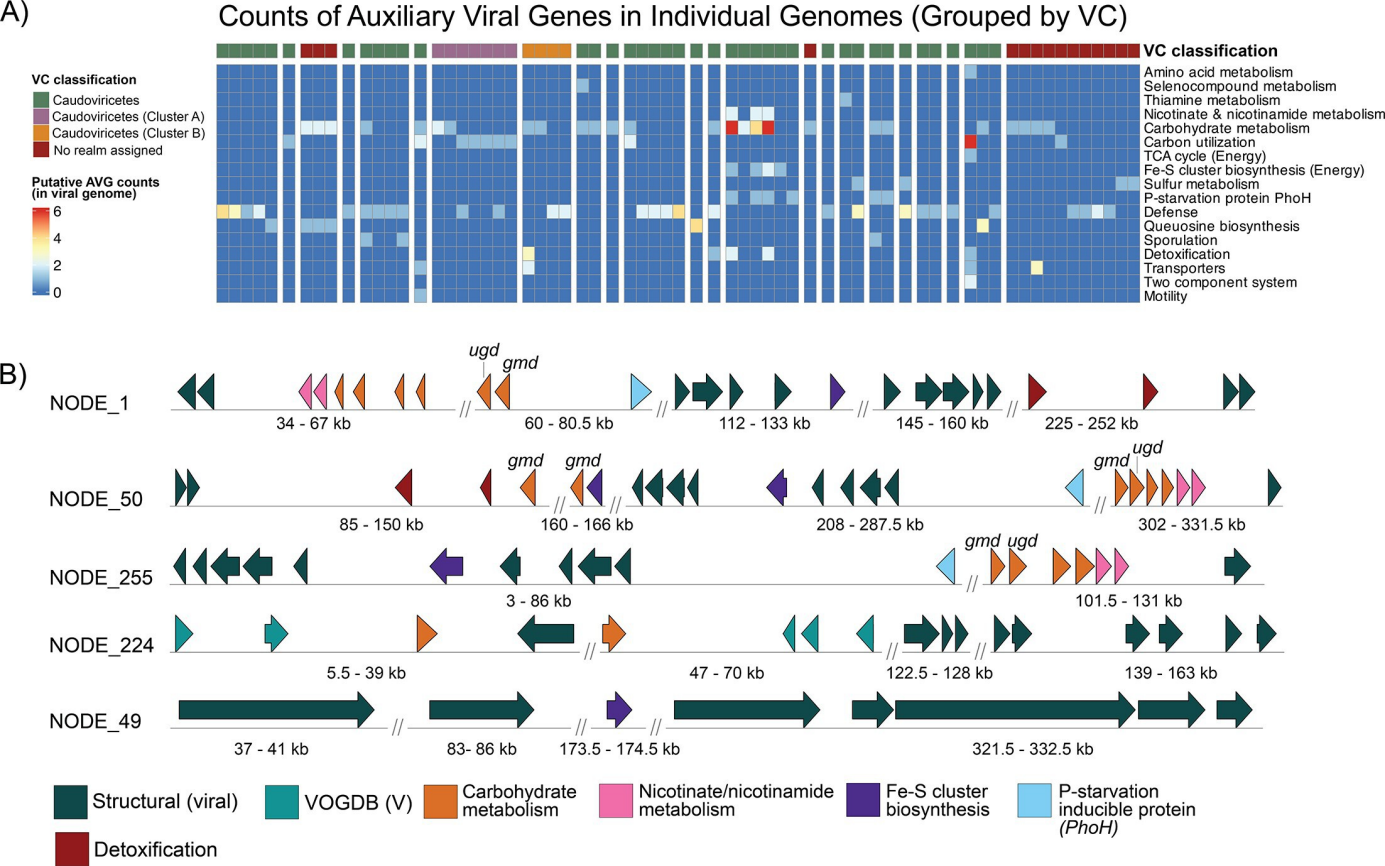
**(A**) Counts of viral accessory genes in individual viral genomes. Columns represent viral metabolic gene counts per contig. The top row indicates vConTACT3 viral cluster (VC) assignments, with spacing grouping viruses within each VC; singletons are shown as individual VCs. Heatmap colors denote gene counts as shown in the legend. (**B**) Gene diagrams of viral metabolic gene distribution across five recovered genomes from vOTU 158, enriched in putative AMGs. Genomes were inspected for flanking viral genes and AMG annotation coverage/identity. Arrows denote genes, and colors indicate AMG categories. The full curated AMG list is provided in Table S8.

Secondary curation of baseline DRAM-v AMG summary hits (212 genes) was performed to eliminate viral life cycle and host-associated genes from AMG candidates. This yielded 72 putative AMGs and 70 accessory (moron) genes associated with defense against mobile genetic elements (87). The majority of AMG candidates and accessory defense genes we identify are from viruses in fumarole biofilms—only one accessory defense gene and one AMG candidate are observed in viruses assembled from non-thermal soil. Overall, 7% of vOTUs (*n* = 27) identified from fumarole metagenomes contain AMG candidates. These low AMG frequencies have been observed in other geothermal systems, such as hydrothermal vents (13). The majority (74%) of AMG-carrying vOTUs are classified as Caudoviricetes, and the remaining vOTUs with AMG candidates cannot be assigned to realms (Fig. 5). Five vOTUs contain AMG candidates related to energy metabolism (boosting ATP-generation). Three of these vOTUs encode NifU-like IscA (scaffold and iron carrier), which is a gene involved in Fe-S cluster biosynthesis. One vOTU contains AMG candidate gene lactate dehydrogenase (a reversible enzyme that converts pyruvate to lactate to generate NAD+ for glycolysis, or converts pyruvate to lactate, feeding the TCA cycle). Another vOTU contains putative AMG succinate dehydrogenase cytochrome b558, which, to our knowledge, has only been described as an AMG in the giant virus family Mimiviridae (88, 89).

The majority of putative AMGs identified in fumarole viruses encode transporter-related gene products (Table S8). Transporter types include: divalent metal transporters, RND efflux pumps, ABC-transporters, and major facilitator superfamily (MFS) transporters. Ten fumarole vOTUs contain AMG candidates that can be linked to cellular detoxification from heavy metals, such as metallothioneins, Zn2+/Cd2+ exporting ATPases, and heavy metal efflux proteins, including transporter TolC. The TolC transporter functions with the efflux of transition metals, including first-row transition metals, which are present in basalt (90). The transporters observed in fumarole viruses have also been described in AMG candidates from viruses in hydrothermal vents and heavy metal-enriched soils with similar elemental composition to basaltic surfaces described in the ERZ volcanic system (2, 20, 91). Other fumarole virus transporters are protective proteins (such as Fe–Mn superoxide dismutase) that neutralize reactive oxygen species (ROS), which can also be generated by metal-induced cellular stress. Fumarolic non-transporter AMG candidates (such as rubrerythrin) also neutralize ROS, which usually confers host survival in microaerophilic or anoxic environments (92).

Other AMG candidates relate to nutrient deficiency. One vOTU contains sporulation maturation protein CgeB, which is sometimes observed in Bacillus subtilis prophages where it is part of the Cge operon that modifies the surface properties of the spore (93). One putative vOTU contains a carbohydrate ABC transporter permease (trehalose transport system permease protein SugB), which is involved in trehalose recycling within the cell (94). One fumarole vOTU (prophage of *Gloeobacter kilaueensis*) encodes a gene highly similar to polyhydroxybutyrate depolymerase (phaZ). Polyhydroxybutyrate depolymerase is an intracellular enzyme that breaks down polyhydroxyalkanoates (PHAs) or polyhydroxybutyrate (PHB) biopolymers, which can be used as carbon sources. Eight unique vOTUs contain AMG candidates related to carbon utilization. Two Caudoviricetes vOTUs contain five different carbon utilization genes that could possibly be involved in PHA biosynthetic pathways, converting short-chain carboxylic acids (such as acetate, propionate, lactate, and butyrate) into PHA (95). Alternatively, these five genes could be involved in beta oxidation of fatty acids, which have been described in the giant virus family Mimiviridae (96). The presence of these five AMG candidates in Caudoviricetes, as observed in two fumarole Caudoviricetes vOTUs, is unusual because Caudoviricetes hosts are prokaryotic, while Mimiviridae hosts are eukaryotic (97). Another putative AMG contained within one of these Caudoviricetes vOTUs is gene 2-methylcitrate dehydratase (prpD), which catabolizes the third step of the methylcitrate cycle for propionate catabolism and has been described as an AMG in hydrothermal vents (98). Host linking with experimental verification of transcriptional activity and gene product expression is necessary to ascertain if these putative AMGs are involved in beta oxidation of fatty acids and lipid catabolism pathways or PHA biosynthesis pathways.

#### Accessory vOTU genes may confer resistance to non-self viruses

In addition to AMG candidates, 32 vOTUs (8% of fumarole-derived vOTUs) contain virally encoded defense genes, which can potentially provide antiviral defense for the microbe (increasing host fitness) or facilitate superinfection exclusion against other viruses. Seventy-eight percent of the defense-containing vOTUs (*n* = 25) were classified as Caudoviricetes, and the remaining defense-containing vOTUs (*n* = 7) could not be assigned to a realm classification. Queosine biosynthesis genes (7-Deazaguanine modification) and (cytosine-5)-methyltransferase (dcm) were initially flagged by DRAM-v as metabolic AMG candidates. However, they were manually reclassified as defense-related because that is more likely reflective of functional role (99, 100).

Fumarole viruses contain a range of defense and anti-defense related genes: anti-CRISPR, anti-Thoeris, and 7-Deazaguanine modification genes were annotated by DRAM-v. Virally encoded toxin/antitoxin (TA) genes from type I to IV systems were observed in ~5% of total fumarole vOTUs (*n* = 18) (Table S8). For most of the vOTUs (*n* = 10) containing TA systems, both toxin and antitoxin genes of the same system type are positioned with no more than five genes between components, suggesting that at least some fumarole viruses encode functional TA systems. Two vOTUs contain CRISPR-associated proteins. One vOTU (vOTU 189) does not have an identified host and encodes a minimal type I-C CRISPR-Cas Cascade system, which contains only three unique Cas proteins: Cas5d, Cas8c, and Cas7. This minimal Cascade encodes an RNA-guided multi-subunit CRISPR-Cas surveillance complex that targets foreign DNA for destruction (101).

A different vOTU (vOTU 212) contains a minimal type IV system (genes Cas6, Csf3, and Csf2) and an unassociated CRISPR array. Type IV systems are primarily encoded by plasmids and more occasionally observed on prophages (102). Type IV CRISPR systems are enigmatic in mechanism and usually lack a CRISPR array. They are hypothesized to modify existing CRISPR-Cas activity of hosts that already contain CRISPR systems (103). Accordingly, vOTU 212 is targeted by spacers within a Chloroflexota (class UBA6077) MAG that contains both type IA and type IE systems (21). In addition to the type IV effector proteins, vOTU 212 encodes a CRISPR array. The maintenance of both an array and type IV system (which characteristically lack associated CRISPR arrays) (104) is suggestive of inter-viral competition that has resulted in the use of CRISPR as a method for viruses to prevent superinfection exclusion (105).

## DISCUSSION

### Fumaroles contain novel and diversifying viruses

Our approach to viral identification that utilizes sensitive, profile-based methods (35) for annotation of structural viral gene components reveals novel viruses from fumarole biofilms and volcanic soil. Our approach identifies viruses using amino acid sequences encoding viral structure components (capsid-associated and packaging genes), which are highly divergent (39). Our recovery of phylogenetically novel viruses from biofilm metagenomes and our subsequent careful manual verification support our workflow, which utilizes remote homology protein prediction for identifying structural virion components. This can, in turn, be used for the identification of viruses in a high-throughput processing pipeline with existing tools (106) without extensive false positives (Table S4).

Our approach yields recovery of highly novel viruses (30), which we find to be sometimes highly abundant relative to other viral populations (Fig. 1). This is predictive of either functional significance for these novel and abundant viruses or represents significant population dormancy (8). Because viruses do not all share a single gene that is common to all species (107), there is no way to confidently place all viruses of microbes on a single phylogenetic tree of life. Thus, it is not possible to phylogenetically estimate viruses that cannot be classified into an existing realm (108). Our assessment of viral diversification for these “no-realm” viruses relies on gene-sharing network analysis, which provides a method for comparison of genomic distance between viruses that cannot be otherwise taxonomically placed. While uncertainty in phage genome completion (vOTUs of variable length within a cluster) and chimeric or mosaic assemblies can constrain comparisons of viral genome similarity, our utilization of NetworkAnalyzer metric of “closeness centrality” (40) minimizes the effects of incomplete viruses producing erroneous inferences of genomic distance (46). Our observation of subclusters for these highly novel viruses, which are not placed into a realm by vConTACT3 and do not exhibit gene sharing with previously described viruses, is evidence that there may be genomic diversification between these novel viruses. Yet, conclusions about associated viral speciation for these highly novel populations are limited by our inability to conduct phylogenetic estimation without realm classification.

The majority of recovered viruses are from the Caudoviricetes class. This could possibly be a result of detection facilitated by the high representation of Caudoviricetes genomes within databases of known viruses (109). As a result, our conclusions about evolutionary patterns in other viral classes are limited by the low genomic diversity of recovered fumarole vOTUs from the Laserviricetes, Tectiliviricetes, and Malgrandaviricetes classes. Even though Caudoviricetes are the most characterized phage class, our identification of fumarole viruses significantly expands the diversity of the class. Out of the 179 Caudoviricetes vOTUs included in phylogenetic estimation, 86 fumarole vOTUs belong to two previously undescribed order-level clades within the Caudoviricetes class (Fig. S2). The 93 other fumarole Caudoviricetes vOTUs have varied levels of relation to previously described viral families. Phylogenetic estimation exhibits that many of the fumarole-derived Caudoviricetes vOTUs are related to families that are primarily incertae sedis within the Caudoviricetes class, such as *Hodgkinviridae*, *Peduoviridae*, *Zobellviridae*, and *Saffermanviridae* (Fig. S3). This suggests that the inclusion of novel viruses may help resolve the phylogenetic positions of problematic taxa within the Caudoviricetes class and enable estimation of clades that are not previously evident. Our observation of distinct Caudoviricetes fumarole vOTUs, which have close phylogenetic relationships (Fig. 2), suggests that not only genomic diversification but also speciation could be occurring between fumarole Caudoviricetes populations.

### Abundant Microviridae may be a signature of geothermal environments

Microviridae are known to be diverse and globally distributed across a variety of environments (sediment, marine, animal, insect, and humans) (72, 74, 110). Despite this, there has never been a description of a terrestrial environment in which Microviridae is the most abundant viral population or otherwise represents a substantial portion of viral abundance in terrestrial or marine (oceanic zone) environments. Benchmarking with mock communities representative of open ocean marine and freshwater environments is consistent with observations that ssDNA viruses make up a very small portion (<5% total DNA viruses) of total viral content and are much less abundant than dsDNA viruses (74, 111). The other environment in which Microviridae has been described as highly abundant is in deep ocean hydrothermal vents sediments (18, 112–114). It is possible that viruses belonging to Microviridae can be adapted to thermal conditions as an ssDNA virus. Phage phiX174 has been demonstrated to be most stable at 37°C–44°C and is predicted to be favored over dsDNA and RNA phages under thermal stress because of the increased access that ssDNA viruses have to mutational diversity (115). Alternatively, it is possible that Microviridae has the capability to circularize which would enable abundance at higher temperatures (116). However, the ERZ fumaroles with the highest Microviridae abundances (50°C–70°C) are over three times cooler than the sediment of Southwest Indian Ocean hydrothermal vents (temperatures as high as 400°C), where Microviridae has been identified as the most abundant virus (18).

Out of ERZ fumarole viral communities, moderately geothermal and high geothermal biofilms are described as having the highest Microviridae abundances out of all samples. However, mesothermal fumarole biofilms are observed to have high Microviridae abundance (22%°C28%), which is higher than described in other terrestrial and marine environments (Fig. 1). This may be explained by the high abundance of Microviridae in mesothermal fumaroles experiencing rapid fluctuations in fumarole temperature. Mesothermal fumaroles, which harbor high Microviridae abundance, may reflect a lag in the decreasing abundance of ssDNA microvirus abundance as the biofilm communities adapt to lower temperatures. Volcanic activity of the ERZ varies widely across small spatial scales and fluctuates rapidly within short time spans (minute to minute), making it possible that the steam pit mesothermal fumaroles are dying geothermal features, while the ~100 m adjacent steam wall is more geothermally active. Many fumaroles are ephemeral and experience more variation in activity as they near the end of their geothermal life span (117).

Viromics is known to overestimate the abundance of ssDNA viruses, and the perception of high abundance is often a sequencing artifact. Many viromics extraction approaches may utilize Adaptase prior to linker amplification and produce a quantitative bias, leading to erroneous predictions of microvirus abundance (118). Alternatively, many viromics DNA preparation methods for low-biomass viromics may destroy Microviridae DNA (114, 119). Our approach utilizes bulk metagenomics prior to sequencing, which may enable the detection of high-abundance Microviridae. We hypothesize that microvirus diversity is underpredicted in geothermal environments, and temperature may be a selective pressure that favors ssDNA viruses (118). Studies of other terrestrial geothermal systems have not yielded similar observations of dominant Microviridae. However, some of these other systems (such as terrestrial hot springs) are often explored in the context of low-biomass sediment that requires amplification or alternative methods of extraction, which may destroy microviruses (119). Alternatively, these geothermal systems are investigated with methods of viromics that destroy ssDNA viral representation in the sample (110). Fumarole biofilms may not be the only terrestrial system whose viral community contains dominant *Microviridae*, but the high fumarole biofilm biomass, which is utilized for DNA extraction, may reveal this characteristic of thermal viral communities, which is otherwise obscured by methodological decisions that are made when investigating the microbial and viral diversity of other terrestrial thermal systems.

Fumarole *Microviridae* are highly novel, with pairwise genome similarities as low as 40%–60% (Fig. 3), as well as syntenic rearrangement between viruses within the fumarole biofilms and compared to reference *Microviridae* (Fig. S6). However, there is a need for comparative genomic approaches when analyzing highly conserved ssDNA viruses to make conclusions about the degree of novelty introduced with fumarole *Microviridae*. Network analysis with vConTACT3 demonstrates that multiple *Microviridae* form a single subcluster, which is unexpected because clusters usually represent genus-level rank (30, 46). This suggests that environmental *Microviridae* are highly conserved and/or significantly under-described.

### Viruses are widely shared between geothermal features of ERZ fumaroles

There is evidence that viral dispersal has consequences for fumarole microbial evolution. Antagonistic coevolutionary relationships between hosts and viruses are often described through a “Kill-the-Winner” dynamic, where both host and virus, engaging in an active arms race, are abundant relative to other viral and microbial populations (120). Relatedly, viral dispersal is thought to be host-dependent (13) and associated with microbial abundance (15). Despite these expectations, we observe that many of the shared vOTUs are only found in low abundances within samples. For microbes that appear to utilize CRISPR as a defense mechanism and have identified arrays, vOTUs were determined to be part of a “specialized” host-virus pair if there were more than three spacers linking a vOTU to a single microbial host (indicative of antagonistic coevolution). The 14 vOTUs which have specialized pairings with microbial hosts are not found in viral relative abundance over 1% within any sample. This suggests that low-abundance viruses and hosts are still antagonistically coevolving and dispersing. Host-dependence and abundance are insufficient factors to predict the limited dispersal of viruses in fumaroles. However, it should be noted that many microbial taxa do not rely on CRISPR for viral defense, and viral novelty constrains the capability for host identification of 90% of identified fumarole vOTUs (84). Yet, even when simply searching for patterns of concurrent variation between abundances of unmatched vOTUs and hosts, no high-abundance concurrent variation that would suggest an expected “Kill-the-Winner” trend was observed (Table S6).

Most vOTUs being shared between fumarole biofilms is highly unexpected. For hot springs and hydrothermal vents, individual vents and pools are biological “islands” with distinct communities between features (112). Microbial communities of Hawaiian fumarole vents exhibit distinct communities as well (2). In hot spring and hydrothermal vent systems, viruses display a distance decay effect with fewer shared viral populations observed with increasing distance between features (12). The majority of viruses being shared between samples is even more unexpected when the context of phototrophic biofilm matrices is considered (12, 14). Compared to other mediums of geothermal systems (sediment or water), geothermal biofilms with strict spatial stratification are expected to have even less dispersal, with deterministic selection occurring on viruses within the microscale of centimeters within the biofilm (121). This is particularly remarkable considering the context of this comparison for fumarole biofilms to hot spring phototrophic biofilms with similar biogeochemical cycling processes and organismal characteristics (15, 77).

The high viral dispersal that is suggested by many shared vOTUs could be explained by unique dispersal mechanisms of fumaroles that diverge from the methods of dispersal seen in hot spring and hydrothermal vent systems. Steam (groundwater heated by magma) may be a mechanism for viral dispersal (1). Steam may enable biofilms to act as “extremophile collectors” that retain viruses within biofilms (1, 2). This suggests Hawaiian fumaroles in the ERZ have unique ecological and complex biogeographic characteristics with high viral dispersal informing these communities. By elucidating the metabolic and phylogenetic diversity of fumarole viruses, we have identified ERZ fumaroles on the Big Island of Hawaii as a promising and unique early Earth analog environment to investigate viral ecology.

### Active fumaroles host diverse viral communities

Soil has been described as a medium that contains one of the most diverse and complex viral communities with high richness and evenness, suggesting significant levels of viral activity (82). In particular, young volcanic soil is known to harbor incredibly diverse microbial and viral communities as a result of the soil’s nutrient-rich physicochemical properties (122–124). Despite these previous descriptions of high volcanic soil viral richness, we discover that the biofilms of active (thermal and steam emitting) fumarole features contain communities with vOTU richness that is significantly higher than vOTU richness of non-thermal volcanic soil from the Kilauea caldera Big Ell cave (Fig. 4). Furthermore, some of the biofilms also contain high vOTU evenness that is comparable to the evenness of volcanic soil. Because biofilms with high viral richness and evenness do not contain a specific microbial phylogenetic profile, we suggest that the environmental context of Hawaiian fumaroles as a geothermal system contributes significantly to the overall viral diversity. There is no clear distance decay effect or geographic partitioning of diversity indexes, indicating that localized biogeographic context is significant in explaining viral diversity. The structural properties of biofilms and microbial metabolism within fumarole features (increased nutrient availability through basaltic bioleaching, metabolism of emitted gases, and temperature) may all contribute to fumarole viral diversity.

The statistically significant relationship between temperature and viral richness has not been described previously in a geothermal geologic system other than hot springs (14, 83). The steady increase of viral diversity in fumaroles, which peaks at 50°C and decreases in high geothermal biofilm samples emitting steam (>70°C), provides compelling evidence that viral diversity in some geothermal systems can adhere to a temperature-diversity gradient, and that biodiversity hotspots in geothermal environments are not limited to “Humboldt’s spa” of hot springs (83). It is known that temperature influences structural biofilm properties, regulating processes, such as viral particle attachment and mineral precipitation (125). Indeed, temperature-dependent biomineralization has been described within moderately geothermal ranges (126). These structural alterations could potentially turn biofilms into physical refugia for virion particles, increasing the density of virion particles with immobilization and, in turn, increasing metagenomically identified viral diversity for species richness while potentially increasing species evenness under specific physicochemical conditions that influence biofilm properties (127). The physical proximity of viruses within the biofilms exhibiting thermal-induced structural properties could allow these viruses to act as “gene highways” (20) and facilitate microbial adaptation to the dynamic and harsh conditions of a volcanic system (Table S8). Even though moderately geothermal samples have high viral richness and evenness, and there is a unimodal relationship between temperature and species richness, microbial diversity indexes of samples do not follow the same clear trend (Fig. 4). The decoupling of fumarole virus and microbial diversity could reflect temporal lag and community turnover of viruses, or dormant but inactive viral populations retained at higher abundances than expected within biofilms.

If the viral richness and evenness within biofilms are representative of active viral populations, this may facilitate increased opportunity for viral competition while increasing the fitness conferred by superinfection exclusion (104, 105). This would contribute to explaining diverse and frequent genomic observations of TA systems (encoded in ~5% of fumarole vOTUs) and other defense-related proteins in fumarole viruses (Table S8).

### *Gloeobacter* prophage may increase host fitness

The steam pit sample site is dominated by *Gloeobacter*, despite the close proximity and presence of other oxygenic phototrophic Cyanobacteria (21), which are faster-growing and photosynthetically efficient compared to *Gloeobacter* species which contain reduced photosystems (128). Two vOTUs (Fig. S8) identified as *Gloeobacter kilaueensis* prophages by global alignment to *Gloeobacter* MAGs (21, 129) are highly abundant and co-vary with *G. kilaueensis* in abundance for all but one of the biofilm samples dominated by *G. kilaueensis* (Fig. 1). The high abundance of both host and prophage suggests that these two vOTUs follow a “Piggyback-the-Winner” pattern of viral ecology (9). Across all vOTUs, these are the only host-matched viruses that directly increase in abundance with corresponding host taxa (Table S6).

The only sample site that contains *Gloeobacter*-dominated biofilms is the steam pit. The steam wall sample site, located ~100 m from the pit, is dominated by other oxygenic phototrophic cyanobacteria and is more exposed to light than the shaded steam pit. *Gloeobacter* lacks a thylakoid membrane and is susceptible to light-induced stress, resulting in metabolic disruption and cellular damage from oxidative stress (129). Our recovery of these vOTUs is the first description of viruses known to infect the *Gloeobacter* genus, which is of astrobiological significance due to its evolutionary role as an ancient cyanobacterium (2, 129). *Gloeobacter kilaueensis* is a highly conserved species with MAGs sharing 99% ANI (21, 129). The presence of multiple vOTUs within steam pit biofilms suggests these prophages introduce genetic variation to the *Gloeobacter* host and facilitate genome evolution of the *Gloeobacter kilaueensis* species. CRISPR arrays in *Gloeobacter* MAGs contain a spacer match to a single vOTU of the genus-rank cluster. The match to a single vOTU within the genus could indicate reduced reliance on CRISPR as defense against the prophage or that the host no longer benefits from defense against vOTUs as they diversify in close proximity without geographic partitioning.

In addition to susceptibility to oxidative stress, *Gloeobacter*’s energy inefficiency makes its high relative abundance in biofilms highly unexpected. All membrane-based energy metabolism occurs within a single layer of the cytoplasmic membrane, restricting surface area and metabolic rates. *Gloeobacter*’s reduced photosystems, light sensitivity, and inefficient RuBisCO (slow carboxylation rate) also limit growth of the genus compared to other oxygenic *Cyanobacteria* (130). Energetically, it is possible that the putative carbon utilization AMG polyhydroxybutyrate depolymerase (*phaZ*) confers host fitness for *Gloeobacter*, contributing to its dominance in steam pit biofilms and optimizing host carbon utilization. Polyhydroxybutyrate depolymerase is an intracellular enzyme that breaks down PHAs or PHB biopolymers. The ability to utilize PHB biopolymers may provide a mobilizable reserve of carbon and energy to *Gloeobacter*, enabling efficient resource management (granules storing nutrients without altering internal osmotic pressure or loss of compounds through leakage) and increasing prophage-mediated *Gloeobacter* survival over other competitors. The vOTU containing the *phaZ* AMG candidate is doubled in abundance compared to the other *Gloeobacter* prophage vOTU, suggesting that there is a comparative fitness benefit to the prophage maintaining the putative AMG.

There is evidence of the Gloeobacter prophage evading host defenses. The presence of what appears to be a self-targeting spacer shows that CRISPR systems of the host may prevent prophage activation or lytic activity (Table S7). Alternatively, the prophage may carry an anti-CRISPR protein that inhibits host CRISPR-Cas systems (*Gloeobacter* MAGs contain both type I and type III CRISPR-Cas systems). The biofilm sample observed, which had both a high abundance of *Gloeobacter* (21) and a low abundance of the *Gloeobacter*-linked vOTUs, was collected from a sunlight-exposed area, with a lower light intensity than the steam wall sample site. High-light stress in exposed biofilms may compromise *Gloeobacter* cells with metabolic disruption or cellular damage, destabilizing prophage maintenance and reducing their relative abundance in the sample (Fig. 1). Oxidative stress could also trigger prophage excision and loss (131). The substantial decrease of prophage content in biofilms despite the host remaining abundant suggests that light-induced stress in Gloeobacter may limit lysogeny (consistent with “Piggyback-the-Winner” models), influencing viral turnover and potentially altering persistence of lysogenic viral populations.

### Accessory viral genes may aid adaptation to basaltic surface and resource limitation

Biofilms contain the majority of putative AMGs. Of identified fumarole vOTUs, only 7% of fumarole vOTUs contain AMG candidates, which is consistent with observations of AMGs as rare in other geothermal environments, such as hydrothermal vents (13). However, more samples with broader geographic distribution are necessary to make conclusions about AMG prevalence in systems with high microbial endemism anticipated (13, 15, 20, 112). The presence of diverse AMG candidates seen within vOTUs suggests that these viruses may function as a genomic reservoir for microbial hosts and facilitate their persistence in adverse and nutrient-limited conditions (Table S8). Basalt-hosted microbial communities in hydrothermal vents are known to engage in bioleaching with enzymatic dissolution of substrate in hydrothermal vents, releasing metals in the process (20, 132). The presence of ten different vOTUs with putative AMG involved in heavy metal detoxification would suggest that viruses play a significant role in facilitating microbial adaptations to high metal concentrations released from the dissolution of basalt (Fig. 5). This explains the presence of detoxifying metal efflux and ROS neutralization putative AMGs, which are often seen in hydrothermal vents and heavy metal contaminated soil where similar processes may occur (18, 90). While most of these transporters are predicted to facilitate heavy metal resistance, some fumarole AMG candidates likely confer fitness for the host in other ways. MFS transporters may alternatively play a role in conferring antibiotic resistance for the host, while biopolymer transport genes are also observed.

Other AMG candidates increase host fitness in resource-limited conditions, primarily with genes that participate in carbon utilization pathways. PHA/PHB metabolism in particular may play an important role in microbial response to adverse conditions. Polyhydroxybutyrate depolymerase is an AMG candidate in a *Gloeobacter* prophage vOTU, which is highly abundant in steam pit samples (Table S8). Two other vOTUs contain carbon utilization putative AMGs, which could be involved in either beta oxidation of fatty acids or PHA biosynthetic pathways (conversion of short-chain carboxylic fatty acids). In resource-limited conditions with nutrient depletion, PHA and PHB utilization could provide microbes the ability to sequester and mobilize carbon and energy sources, aiding survival in dynamic and volcanism-dependent physicochemical conditions of fumaroles. Chitin-degrading genes (chitinase and chitosanase) are contained by three different fumarole vOTUs and are AMG candidates. Chitinase has been described as an AMG in prophage-encoded chitinase within hydrothermal vents, where the AMG is involved in substrate-dependent lysis-lysogeny switches of viruses. It is known that carbohydrate degradation capacity is a driver of microbial community structure in hadal zones, offering a possible explanation for how these putative AMGs could confer host fitness in nutrient-limited and dynamic environments, such as fumarole biofilms (131). Putative AMGs related to nutrient limitation suggest viral AMGs confer host fitness with AMGs related to resource acquisition and scavenging (4, 98, 133).

Sporulation-related AMG candidates may facilitate survival in adverse conditions via dormancy, which has primarily been observed as an AMG in low oxygen zones, heavy metal-contaminated soil, and sub-freezing environments (91, 134, 135). These putative AMGs may help their host by facilitating microbial sporulation when conditions are resource-limited until nutrients are available for host reactivation. The selection for microbial survivability under resource limitation may incentivize the acquisition of viruses with these AMG candidates. However, identification of viral metabolic genes is constrained by sequence length (contigs under 10 kb). Biochemical characterization or transcriptomic changes provide evidence for confident AMG identifications and should be performed for any structurally novel predicted AMGs. This is particularly true for CAZyme genes, which are poorly conserved on a structural level (61).

## Data Availability

All raw data used in this study have been deposited in the European Nucleotide Archive (ENA) under project accession number PRJEB52128. Phylogenetic IQ-Trees, viral candidate contigs, and final viral contigs are available on Zenodo at DOI: https://doi.org/10.5281/zenodo.19378070. Scripts to run analyses and the viral identification pipeline custom scripts are available at https://github.com/pia-sen/Hawaii_fumarole_viruses.

## SUPPLEMENTAL MATERIAL

Fig. S1 spectrum.01567-26-s0001.tif vOTU identification by tool.

Fig. S2 spectrum.01567-26-s0002.tif Phylogenetic tree with fumarole viruses which are part of order-level novel clades highlighted as green and red nodes (Clade 1 and Clade 2, respectively).

Fig. S3 spectrum.01567-26-s0003.tif Class level Caudoviricetes phylogenetic tree with known viral families denoted in color.

Fig. S4 spectrum.01567-26-s0004.tif Class level phylogenetic estimation of Tectiviricetes and Laserviricetes fumarole viruses (colored in red).

Fig. S5 spectrum.01567-26-s0005.tif Malgrandaviricetes class level phylogeny demonstrating poorly resolved clade of fumarole Microviridae viruses and phiX174.

Fig. S6 spectrum.01567-26-s0006.tif Comparative genomics of fumarole Microviridae and RefSeq Bullavirinae (phiX-like) viruses: showing syntenic variation and gene sequence conservation.

Fig. S7 spectrum.01567-26-s0007.tif Diagnostic plots for non-linear regression of temperature and vOTU richness and evenness.

Fig. S8 spectrum.01567-26-s0008.tif Comparative map of three fumarole vOTUs representing first description of Gloeobacter phages.

Supplemental tables spectrum.01567-26-s0009.xlsx Tables S1 to S9.

ASM does not own the copyrights to Supplemental Material that may be linked to, or accessed through, an article. The authors have granted ASM a non-exclusive, world-wide license to publish the Supplemental Material files. Please contact the corresponding author directly for reuse.
